# Morita Therapy as a Treatment Approach for Adolescent Autism Spectrum Disorder: A Five-Year Case Report Incorporating Inpatient and Diary Therapy

**DOI:** 10.7759/cureus.61750

**Published:** 2024-06-05

**Authors:** Mitsuhiro Nakamura

**Affiliations:** 1 Psychiatry, Yokohama Camellia Hospital, Kanagawa, JPN; 2 Psychiatry, Shinano Mental Clinic, Kanagawa, JPN

**Keywords:** autism spectrum disorder (asd), morita therapy, asia-pacific, child and adolescent psychiatry, psychiatry, school psychology

## Abstract

In 1919, Shoma Morita established Morita therapy, and this method of psychotherapy is widely used in Japan and across the world. With time, the medical indications of Morita therapy have expanded to include not only neurosis and anxiety disorders but other conditions as well. In modern times, Morita therapy has been used to treat adolescentneurodevelopmental disorders; however, it has not been widely covered in the English-language literature. In this report, a five-year course of treatment for a female patient with autism spectrum disorder (ASD) is presented. The patient exhibited dissociation, auditory hallucinations, overmedication, and wrist cutting, leading to multiple admissions to an adolescent ward. Over the treatment course, the symptoms of dissociation, self-harm, and auditory hallucinations disappear. Further, the patient was able to find a way to relate to society that was appropriate for her.

## Introduction

Morita therapy, developed by Shoma Morita in 1919, is a Japanese psychotherapy technique that is now used worldwide for nervousness and anxiety disorders [1-4]. Morita therapy considers fear or anxiety to be a single concept with two facets [1,4]. One aspect represents the fear we broadly recognize as being frightened, whereas the other aspect indicates the positive side of fear, that is, the desire to live. Furthermore, in Morita therapy, anxiety or fear is considered to originate from a desire to live [1]. Morita therapy emphasizes the positive aspect of fear. Based on this view of fear and anxiety, the therapeutic goal of Morita therapy, arugamama, combines the following two approaches [2,4]. The first approach is to view the emotions of fear and anxiety as they are, and the second is to bring out the raw desire behind the fear and anxiety in the real world. To reach the treatment goal of arugamama, the classical Morita approach to therapy is inpatient therapy structured in four layers (isolation rest, light monotonous work, labor-intensive work, and social integration); however, for various reasons, the number of facilities that can conduct inpatient Morita therapy has decreased. Currently, the mainstream approach for Morita therapy is outpatient therapy based on dialogue in combination with a diary [1-4]. Diary therapy was used in Shoma Morita’s original treatment practices, either as a part of inpatient therapy or as correspondence therapy [1-3]. The diary is an important tool in modern Morita therapy. It provides a trajectory of the patient’s behavior and emotional experiences as well as the trajectory of the treatment itself, including the patient’s relationship with the therapist [1,3]. In other words, to achieve the treatment goal, patients are anxious to take action, face obstacles, and seek a different way of doing things. Through the diary, the therapist supports this behavior with empathy and encouragement while simultaneously illuminating the patient’s healthy desire [1-3].

As such, dominant therapeutic approaches now emphasize understanding and nurturing personal *raw desire* or the *desire to live*, using the terms of Morita therapy, alongside patients’ lifestyles [1,3,4]. Moreover, Morita therapy has expedited its scope to encompass a spectrum of contemporary conditions, including those traditionally viewed as incompatible, such as narcissistic disorders and autism spectrum disorder (ASD) [1].

Morita therapy for ASD

During Morita’s lifetime, ASD was not within the scope of Morita therapy’s treatment targets. However, a pivotal moment came with the introduction of the concept of developmental disorders to Japan, spurred by the third revision of the Diagnostic and Statistical Manual (DSM-III) in the United States during the 1980s and 1990s [5]. This provided an opportunity for the expansion of Morita therapy in Japan, ultimately leading to the emergence of clinical practices dedicated to developmental disorders, including ASD, which became a central focus in child and adolescent clinical care. As the global awareness of developmental disorders increased, Morita therapy was tailored to accommodate individuals with ASD, and this adaptation became publicly available.

Kora, a disciple of Morita, puts forward a distinctive perspective on this development [6,7]. In contrast with Morita’s emphasis on personality and predisposition as underlying mechanisms of neurosis, Kora introduces the concept of *inadaptability anxiety*. This concept refers to the tension between the self and the surrounding environment, as characterized by the individual’s struggle to accept themselves in their immediate context. During adolescence, individuals with ASD often exhibit distinctive types of interactions with their surroundings, characterized by challenges in communication, fixation on specific interests, and difficulties in social interactions. These aspects tend to influence these individuals’ distinctive forms of adaptation to life, which are often described as idiosyncratic or, at times, maladaptive. However, earlier studies investigating Morita therapy in the context of the interplay between the self and the environment have primarily focused on its application for environmental adaptation [8].

In Okubo’s study, the focus is on the treatment of the anxiety disorders associated with ASD. Research suggests that this treatment is particularly effective when the severity of ASD is mild and when patients can diligently record their anxiety experiences in a diary [9]. Child and adolescent psychiatry wards, such as the author’s workplace, face distinct challenges. In such wards, adolescent ASD can be marked by severe symptoms, including suicide attempts, drug overdoses, listlessness, self-harm, and dissociation [10,11]. Treatment of individuals who have both severe ASD and other psychiatric symptoms using Morita therapy has not been documented to date. It is crucial to gain insight into comprehending and addressing the complexities of ASD from the perspective of Morita therapy, incorporating techniques such as diary therapy. The potential growth-enhancing effects of these interventions remain largely unexplored. In this paper, I address this gap by presenting the treatment journey of Morita therapy for a severe case of ASD involving multiple hospitalizations. I explore the case within the therapeutic framework of Morita therapy to provide valuable insight and understanding. Few psychotherapies prioritize the practical application of therapy within an individual’s daily living environment as extensively as Morita therapy does. It is my conviction that investigating the treatment progression for ASD through the lens of Morita therapy will facilitate its widespread acceptance within the child and adolescent psychiatry community as a developmental and growth-oriented approach for adolescent patients with ASD. This study was performed by the Helsinki Declaration and was approved by the institutional review board of the Yokohama Camellia Hospital. Furthermore, written informed consent was obtained from the participant. The case details have been modified to protect the patient’s privacy without significantly changing the context.

## Case presentation

Patient characteristics

The patient was a 17-year-old female with ASD or Asperger’s syndrome in ICD-10 at the time of initial examination, and her chief complaint was a desire to die. There was no family history of note.

Life History

The patient, born at full term, weighing 3,200 g, was the older of two children. She lived with her father, a researcher in his 40s at her birth, and her mother, who was employed part-time. Her sister was two years younger than her. Developmentally, she reached milestones within the typical range, including first words and basic skills. As an infant, she exhibited minimal nighttime fussiness and was generally easy to care for. By age three, she already read both hiragana and katakana, which are the most basic types of Japanese characters and are usually studied in the first year of elementary school. However, in kindergarten, she did not excel in ball games or sports. This led to a preference for solitary activities. During her elementary school years, she exhibited a deep passion for reading. However, by fifth grade, she began to experience neglect and bullying. Attempts to seek support from family or teachers often resulted in blame, leaving her feeling misunderstood and unsupported. She endured occasional physical abuse from her father. She believed that her sole path to escape her local school was to pass the junior high school entrance exam. In pursuit of this goal, she diligently attended a cram school, dedicating herself to her studies. She gained admission to a private junior high school in X-5. Despite hopes for a more positive experience, she felt alienated among peers whom she perceived as academically superior. Struggling to engage with her classmates, she experienced feelings of displacement and loss of appetite, leading to school absenteeism. In addition to intermittent attendance, she grappled with bouts of dizziness and difficulty waking up, ultimately leading her to transition to a correspondence high school for her high school education.

History of Present Illness

Upon her enrolment in a correspondence high school in her 15 in year X-2, she managed to attend school regularly without significant interruptions. However, as time progressed, she questioned herself: “What am I achieving despite all my dedicated studying?” This internal reflection triggered dizziness and a recurrence of symptoms from halfway through the first year. Seeking help, she was admitted to a psychiatric clinic after experiencing suicidal thoughts and resorted to cutting her wrists as a coping mechanism after entering her sophomore year of high school in year X-1. Although she was prescribed antidepressants such as sertraline, escitalopram, and duloxetine, her condition deteriorated. In the year X, she began to experience auditory hallucinations of swearing in the voices of her mother and classmates and hearing her thoughts as words, coinciding with mood swings and anxiety. After receiving the unsettling news from the hospital that she might have a developmental disorder, she was enveloped by a profound sense of despair. She feared that her diagnosis might prevent her from pursuing a desired career in design or counseling, intensifying her thoughts of self-harm and suicidal ideation. Consequently, in March of year X (her second year of high school), she was referred to us for hospitalization.

Predictive/Working Diagnosis

During the initial examination, the patient’s tendency to tilt her head while responding to questions and her limited speech usage revealed a distinctive mode of communication. Reflecting on her upbringing, she realized that her parents, particularly her father, perceived her as unresponsive from an early age, struggling with the challenges of raising her. Her recollections also highlighted hurdles in social communication during her preschool years, including disinterest in playmates, peers, and subjects that failed to capture her attention. The WAIS (Wechsler Adult Intelligence Scale)-R administered during her hospitalization revealed a verbal IQ of 112, a performance IQ of 98, and a Full-Scale IQ score of 108. It should be noted that her performance IQ lagged significantly behind her verbal IQ, with an examination of subtest items revealing exceptionally low processing speed. Despite cognitive variations, no pervasive developmental delay was observed, confirming a diagnosis of Asperger’s syndrome.

She experienced frequent episodes of hallucination, likely stemming from early parental relationships of trauma and post-elementary school bullying. These distressing experiences left her perpetually stressed, disconnected her from her sense of self, struggling to process her emotions, and ultimately falling into a chronic depressive mood. These symptoms manifested as dissociative auditory hallucinations, occurring alongside suicidal ideation.

Initial phase of treatment progress

First Hospitalization (March to July Rear X)

During her initial hospitalization, the medical team adjusted the patient’s medication to alleviate her symptoms, introduced interaction programs in the adolescent ward, and initiated a process of self-discovery and future planning. This became the primary focus of her hospitalization. She was voluntarily hospitalized on the day of arrival. Shortly after admission, she entered her third year of high school, feeling concerned about her school attendance and future career path. However, upon consultation with her homeroom teacher and her mother, they decided that she would continue her education at a moderate pace while receiving hospital care. She was able to attend school approximately one day per week from her hospital ward. Given her ongoing depressive mood and suicidal ideation, a treatment plan was initiated, including a daily prescription of 3 mg aripiprazole. Disclosure of the Asperger syndrome diagnosis to her and her parents met with reserved acceptance. When the dose of aripiprazole was increased to 12 mg, her depressive mood gradually improved, allowing her to concentrate on occupational therapy. During this period, she continued to maintain a limited degree of communication with fellow patients, she and her family engaged in extensive consultations with the hospital on the prospect of spending nights at home, which is often the case in voluntary hospitalization, compared with compulsory hospitalization, in Japan. Initially distressed upon returning home, expressing frustration that her family failed to understand her and recalling past painful experiences, family interviews were conducted to address these concerns. Emphasizing the importance of refraining from scolding or criticizing the patient and encouraging her growth, her family gradually made her more at ease with staying overnight at home, and she eventually achieved stability. This progress facilitated her discharge from the hospital in July of year X, during her third year of high school.

Outpatient-Inpatient Rehabilitation-Outpatient (July Year X to January Year X + 2)

Following her discharge, the patient continued regular visits once or twice a month, showing promising progress. She continued schooling at her own pace, attending classes two or three times a week, reporting her experiences, participating in overnight school events, and even joining school trips. However, during medical appointments, when confronted with complex emotional and future-oriented questions, she responded with a pensive *Hmm*, as before.

She opted to attend a junior college after graduation and began to attend a cram school. However, her demeanor conveyed reluctance and anxiety. Minor disputes with her parents over trivial matters triggered a recurrence of depression. The addition of olanzapine 2.5 mg as pharmacotherapy for anxiety and depression had no significant effect. Her dwindling confidence in her ability to spend the year-end and New Year holidays with her family, after a period of increased contact with them, prompted a discussion with her parents. A collective decision for her short-term hospitalization was made to enable her to address these challenges. She thus embarked on her second hospitalization, this time for a restorative period of 10 days, beginning on December 29, year X. Following her discharge, she passed the junior college entrance exam and began studies in the English department in April of year X + 1. However, she grappled with a sense of reluctance toward studying. In the early stages of her enrolment, she managed to attend classes for about a month. However, she found it challenging to make friends and felt uncomfortable seeking assistance from her classmates and teachers when she encountered difficulties, such as in understanding how to submit assignments. The drug therapy, an increase in the dose of olanzapine to 5 mg, was ineffective. As May progressed, her absences became more frequent, leading ultimately to a leave of absence in June.

The patient’s aspirations to achieve top grades and transfer to a four-year university were left unfulfilled, and this exacerbated her depressive symptoms. The auditory hallucinations, which had calmed down, began to recur. The hospital, in addition to making adjustments to her medication by starting a small dose of fluvoxamine for depression and added up to 50 mg, recommended that she take advantage of school counseling as a safe space where she could discuss her concerns while attending junior college. These counseling sessions, which took place twice a month, provided regular support, and from September onward, her visits to the hospital fell to once per month. Gradually, her situation improved, and she was able to attend junior college approximately three times a week.

Nevertheless, in January year X + 2, her depression, auditory hallucination and recurring suicidal ideation intensified during increased family contact over winter vacation. This led her to contemplate hospitalization again, leading to additional hospital visits on an as-needed basis.

Despite her reticence in the examination room, her exceptional language skills allowed the hospital staff to effectively investigate the emotions that underpinned her suicidal ideation and depression. They encouraged her to begin by expressing her feelings through diary writing. She complied with this suggestion and began to meticulously detail her concerns regarding her family, her struggles with interpersonal communication, her anxieties concerning her sexuality, and her academic insecurities. In response to these revelations, the hospital proposed inpatient treatment utilizing diary writing to address the emotional underpinnings of her suicidal thoughts, depression, feelings of impasse, and the disparity between her ideal and actual selves. Upon receiving this proposal, she was voluntarily admitted for the third time in January year X + 2.

Latter half of treatment progress

Third Hospitalization

The hospital adopted a novel approach that prioritized her living within a ward program using her diary as a tool for emotional introspection and self-discovery, rather than solely targeting specific symptoms like suicidal ideation or depression.

In her first diary entry (Diary 1), she described interactions with unfamiliar people as *poisonous* and conversations with strangers as *deadly poisonous*. The doctor acknowledged her interpersonal relationship challenges and admired the sensitivity shown in her self-expression. In the therapeutic alliance thus built, which involved a shared exploration of her path toward *detoxification* from this *poison*, the doctor humbly expressed gratitude: “Thank you for enlightening me.” This exchange revealed the depth of the patient’s struggles, which had not been fully grasped by her social circle or previous medical professionals, highlighting the doctor’s empathetic response as pivotal.

In alignment with her diary therapy in Morita therapy, the doctor provided weekly feedback, addressing the actions and emotions articulated by the patient in her diary, based on her genuine desires. When the patient expressed anxiety about interpersonal relationships in her diary, she was reminded that such feelings are a natural aspect of human existence, and she was guided to recognize the importance of coexisting with them. The doctor and the patient worked together on techniques to navigate this process effectively.

Diary 1

January X+2 first day of hospitalization 7:30 pm to 8:30

​​​ *As I embark on my third hospitalization, the prolonged gap since my second stay-more than a year, to be precise-has ushered in a plethora of new faces to the hospital environment. Engaging in conversations with these unfamiliar people leaves me feeling exceptionally drained. If we were to draw a parallel with the states of game characters in video games, it’s akin to them becoming “poisoned” when conversing with someone they haven’t met before and “deadly poisoned” when interacting with individuals they’re encountering for the first time (comments: Thank you for making it clear to us). In the past, I used to converse with people without feeling fatigued. However, since my middle school years, I’ve felt ensnared in a “poisonous enchantment.” When I was in my first year of junior high school, a friend said to me, “Everyone is saying bad things about you,” and for the first time I thought to myself, “Oh, I was spending my time thinking that I didn’t want everyone to hate me, but the truth was... everybody hated me.” Maybe I’ve been under a spell ever since that day. Moreover, even during my time in elementary school, I was no stranger to people around me uttering negative remarks about me. However, then, I thought, “People who say bad things are low-level.” I yearned for guidance to break free from this toxic cycle. With each hospitalization, a persistent thought lingers: “Even in the confines of these hospital walls, my symptoms persist unabated.” Rest is undoubtedly crucial, but solitude does not appear to be the remedy I seek. The question looms large: How can I truly cure this affliction? If the solution were apparent, surely it would have manifested by now. Yet, the persistence of my condition implies that I remain in the dark. Despite this ongoing struggle, my determination to uncover a remedy for my ailment remains unwavering (comments: Little by little, to find a way of life that suits you.). I am fortunate to have a supportive environment that accommodates my recurring hospitalizations. Surrounded by the unwavering support of numerous individuals, I understand that my life is not solely my own. I am determined to give my utmost effort.*

The patient expressed sorrow regarding the disparity between her current state and the self she remembered from preparatory school. However, through empathetic understanding, we discerned that this apparent negativity stemmed from her profound desire for a life with greater promise.

At times, the patient candidly expressed her reluctance to engage with her junior college assignments or attend classes. Embracing her hesitation, we offered acknowledgment that such feelings were valid, and we supported her in taking manageable steps forward. I encouraged her to attend her classes, and to our delight, she showed her determination by diligently completing and submitting her assignments, revealing a strong work ethic. During spring break, she wholeheartedly embraced her passions for anime, pop culture idols, and smartphone games. We respected her interests and wholeheartedly encouraged her participation in events related to the anime she was interested in. In this manner, amidst a whirlwind of emotions-fear, desire, unfulfilled longing, and an unwavering will to live-we assisted her in focusing on her particular interests.

Although the school administration expressed concern about her ability to attend while she was at the hospital, there were no instances of suicide attempts or self-harm in the hospital ward or within the therapeutic setting with the use of a diary. Given this positive progress, we approached her with the proposal that she return to school and requested her cooperation in doing so. We arranged for her to meet with her homeroom teacher, and since April, she has been attending school approximately twice a week. Her interest in her sociology seminars flourished, allowing her to regularly attend and engage in thought-provoking discussions in that context. Further, the ward program helped her form connections with peers who were her age. In her adolescent group therapy sessions, she fearlessly opened up and spoke of her concerns regarding her sexual orientation and not knowing whether she was lesbian or straight. To her delight, the other members of her group offered sympathy and wholehearted acceptance. This experience, validated by both her doctor and fellow ward residents, significantly enriched her self-awareness and self-acceptance.

The patient’s medical examinations and diary entries spanned a broad spectrum, from the intricacies of her anxiety symptoms to the dynamics of her daily life within the ward, school attendance, completion of assignments, and even self-esteem, including her exploration of her sexuality. Gradually, she began to delve deeper into her sense of self. In May, she was discharged from the hospital after spending 102 days as an inpatient. During this admission, diary therapy, occupational therapy, and adolescent group therapy were provided, and medications remained unchanged: aripiprazole 12 mg, olanzapine 5 mg, and fluvoxamine 50 mg. Following her discharge, the patient maintained regular weekly visits to the outpatient clinic, where we diligently monitored her progress through her diary entries. Throughout her hospitalization, she remained dedicated to exploring her actions and emotions, aligning with the principles of “raw desire” central to Morita therapy. We supplemented her diary entries with comments, guiding her towards further growth and self-awareness.

She maintained a stable weekly routine throughout the first academic semester from May to July, and her symptoms remained stable. Her research topic at school focused on sexual minorities, aligning with her personal interests. She actively participated in student clubs organized around sexual minorities and even took on leadership roles in certain events. Her summer vacation was a bustling mix of seminar camps, club summer camps, and working part-time as a box lunch vendor. She vividly captured the essence of this period in her diary, describing it was “the first time in my life that I was able to enjoy a truly fulfilling summer vacation.” During this time, she navigated her interactions with her friends, once considered *poisonous*, in her unique way, maintaining a healthy distance from them. At the same time, by this time, she rarely experienced auditory hallucinations anymore.

At the beginning of the second semester, in September, she expressed a strong desire to excel in her studies, resulting in her attending school for five days per week and increased involvement in club activities during the school festival. She also observed a personal transformation, gradually becoming more adept in responding to inquiries from others. Even during her outpatient consultations, she showed a noticeable improvement in her ability to respond promptly and express herself effectively. Between January and February, fluvoxamine was discontinued and aripiprazole was reduced to 3 mg. After April X + 3, she decided to re-enroll at a four-year university, showing a strong determination to continue her studies in sociology. To prevent a recurrence of the challenges she faced during her junior college years, discussions were initiated regarding her approach to the early days at the university.

However, upon commencing her studies at the university, she felt compelled to establish new friendships, yet encountering difficulty integrating into peer groups, resulting in frequent absences during her first semester. Nevertheless, during the summer break, she expressed the desire to secure a part-time role at an amusement park, which involved donning costumes. Initially, her doctor expressed concern about this position in light of her unique characteristics. However, it became apparent over time that the treatment structure incorporating diary writing provided the necessary support.

Her doctor offered her reassurance, stating, “You can certainly give it a try, but if you find it too challenging, don’t hesitate to discontinue it immediately.” Eventually, she decided to leave her part-time job at the amusement park and began another part-time job at a call center and as a web writer. It became evident that she had a natural talent for writing, excelling in self-expression through words. When her writing abilities were recognized and appreciated, her self-acceptance increased. Around this time, she began to reconcile with her family, particularly her father and spent more time with them. During her second semester at university, she made the effort to attend as many classes as possible.

In addition to her writing pursuits, she maintained stability in her life by balancing part-time work at a restaurant, engaging in online gaming, indulging in her love for anime, and actively participating in a club for sexual minorities. By the time she reached year X + 4, she had harnessed her unique qualities to participate in a range of activities both within and outside the university. She became adept at “fully utilizing her own strengths” without relying heavily on her doctor’s support as she had in the past. At this point, she truly began to feel alive. Olanzapine was discontinued in February X+4.

The therapist gave her homework to describe “What it means to be cured.” This homework assignment was also given at the Morita therapy-based self-help group “Seikatsu no hakkenkai” (Diary 2), but she was not a participant there [12].

Diary 2

My reflections on the process of healing

Let’s delve into the heart of the matter. In my current situation, what I aim to “address” is the tendency to “create circumstances that render life so arduous that I contemplate giving up.” With this in mind, I believe the primary objective of my hospital treatment is to “safeguard against the occurrence of psychologically self-destructive behaviors that could potentially prove fatal.”

Lately, driven by a sense of obligation, I’ve been dedicating myself to specific tasks, despite still grappling with the challenge of studying English, a subject I once detested and struggled with. Nonetheless, I maintain the minimum effort required to earn credits in mandatory courses. I’ve also decided to step away from STK (note: this is the name of a club the patient attended at university, in which members would copy the dances of famous music groups.), choosing to focus on my enduring passion for writing, despite occasional doubts about my proficiency.

In essence, I can confidently declare that I am in the process of healing and recovering from my illness.

Looking ahead, I aim to strike a balance and reduce my reliance on hospitals and school counselors, while seeking additional sources of support and guidance to create a more manageable environment. I deeply appreciate my school counselor, who has been invaluable to me since the fall semester. However, as I prepare to graduate from college and enter the workforce, ongoing counseling sessions can become a significant financial burden, necessitating a reevaluation of my priorities. I aspire to eventually live independently without relying on counseling.

My sentiments toward hospitals bear some similarities to my feelings about counseling. Although I may need to continue visiting to maintain a mental disability certificate to some extent.

Possessing a disability certificate has played a vital role in stabilizing my mental health, and I wish to retain it as a source of strength and support in the future. I acknowledge that this could be perceived as disrespectful to others facing similar struggles in their lives.

(comment: I’m impressed. You are truly remarkable.)

This narrative explains the process of embracing negative emotions associated with personal attributes and areas of weakness, alongside learning to engage with the environment in a manner that is true to one’s self, gradually approaching a state of arugamama, which is a pivotal therapeutic objective within Morita therapy [1,4]

The remaining 3 mg of aripiprazole was discontinued, and the drug therapy phase concluded in July X + 4. A short time later, we discussed ending the hospital visits and diary therapy, and the overall treatment concluded in January X + 5. No symptoms of dissociation, auditory hallucinations, or suicidal ideation had been noted over the past year. The patient has not consulted with us since then. (Please refer to Diary 3, dated December X + 4).

Diary 3

December 31, X + 4

The routine of attending university and classes from Monday to Friday has become second nature to me. After my six-month leave of absence, I’ve managed to build a substantial network of acquaintances among both first- and second-year students. Surprisingly, what was once a daunting task-dealing with tuition assistance in April-has evolved into an eagerly anticipated event.

As I perused my diary to recollect the latter part of X + 3, I realized that I was attending school five days a week during the second semester of that year. Despite the rigorous schedule, I earned fewer credits than I do now. Moreover, I didn’t hold a steady part-time job at that time.

X + 4 has been a year of transformation for me. However, change has been a constant companion in my life, year after year. Since I initiated “Note” (a Japanese-language SNS that mainly uses text) in January, I’ve had the opportunity to connect with diverse individuals. These include a woman in her 30s living with a woman, a world-traveling designer, a K University student creating a picture book about the LGBT+ community, and a creator who is in the process of completing a graduation project centered around me.

Feeling like there was no space for me within the university, I decided to take matters into my own hands, telling myself, “If there isn’t a place, let’s create one.” I took the initiative to establish a club and assumed the role of its president. Interestingly, when I approached the seminar teacher, I had been eager to become the advisor for our club, they responded, “I’ll grant you credit for my seminar.” Inwardly, I couldn’t help but think, “Sir, you didn’t need to say it out loud; it’s more than enough if you just feel that way.”

At the end of X + 3, I was in a relationship with a man, but at the end of X + 4, I am in a relationship with a woman. Rather than categorizing it as “I like women,” it’s more accurate to say, “I like this person.” She celebrated my birthday, and we shared a unique Christmas celebration (on the 26th and 27th due to my part-time job on Christmas Day), and we have plans for a hot spring trip this February. As I ponder how to make my girlfriend’s April birthday special, I can’t help but feel content. This relationship has already surpassed six months, making it the longest one I’ve ever been in, so the prospect of it continuing beyond April brings me immense joy.

I’ve struggled to prioritize self-care, and that challenge persists even now. Consequently, I’ve resorted to making commitments to others in an attempt to motivate myself. I’ve made statements like, “I’ll offer class support on Mondays, ensuring that if I skip university, those around me will be affected.” I’ve also resolved to create a university club, assuming the role of president, with the goal of collaborating with individuals who encounter similar challenges.

Additionally, I’ve entered into a monthly donation agreement with a certified NPO, dedicating 2,000 yen to support refugee children. This not only serves as a financial contribution but also eases my emotional burden when it comes to my part-time job. Also, my girlfriend does her best to tell me to take it easy once in a while. I take a break for my girlfriend. Through such “inventions,” I am trying to become a “normal person.” I think if you have a certain level of good outward appearance, life will be manageable.

My aspiration for X + 5 is to adopt a positive perspective on life’s many facets. I aim to reflect at the year’s end and think, “Despite the challenges we faced, it was an enjoyable year.” There’s nothing wrong with maintaining the status quo.

On a side note, my work schedule involves four consecutive days from the end of the year to January 3rd due to my part-time position at a conveyor belt sushi restaurant. It’s a means of earning a livelihood.

## Discussion

Summary of the treatment course

The preceding case details the progression of treatment for an adolescent patient with ASD who underwent inpatient therapy guided by Morita therapy, ultimately reaching a conclusive stage in their therapy.

During the initial phase of the treatment, my primary focus was on addressing the profound challenges related to the patient’s self-experience, which manifested as overwhelming issues in various facets of her life, including home, school, and other environments. She exhibited symptoms of fragmentation, dissociation, overstimulation, and even auditory hallucinations [10,11,13,14]. To provide necessary support, several interventions were implemented.

First, creating a safe and secure environment was prioritized, at times necessitating hospitalization to temporarily isolate her from challenging surroundings and facilitate much-needed rest. Multiple interviews were conducted with the patient’s family and representatives of her school to gain a comprehensive understanding of her situation.

Recognizing the significance of her interaction with her environment, her occupational activities over her interpersonal relationships were emphasized. In the inpatient setting, occupational therapy to promote her engagement and development was introduced. Additionally, it was ensured that she could continue her schoolwork without subjecting her to the overwhelming demands of the outpatient setting, making the necessary adjustments to support her academic progress. Gradually, a network of professionals who could constantly communicate with her was established. This network extended beyond the hospital staff, encompassing school counselors, forming a comprehensive support system centered around the doctor-patient relationship. As the patient’s hospitalizations increased, her environment began to improve. Before her third hospitalization, we introduced diary therapy, which empowered her to articulate her emotions in her own words and enabled her to cope better with her feelings.

The initial treatment phase resembled a typical clinical trial that involved an adolescent. However, it served as an introduction to the goal of Morita therapy - achieving a state of *arugamama*, allowing necessary actions while maintaining her fear or anxiety [4]. It proved challenging to navigate her raw desires within her fragmented self, but we made concerted efforts to recognize her preferences and sensibilities. Her raw desires were accorded the respect they deserved based on identified elements that she favored or resonated with.

In the latter part of the treatment, with the introduction of diary therapy, significant therapeutic depth was achieved. She had difficulty expressing her emotions on the spot, a common characteristic of individuals with ASD. During our regular office visits, she seldom spoke. However, in her diary entries, she found a unique way to express her emotions, enabling her to better manage and navigate her feelings. The patient’s fragmented self gradually began to coalesce.

Here, her focus shifted to considering how she related her emotions and environments to a given situation, adapting her themes about the circumstances. Beyond the support gained from the therapeutic relationship, her participation in group experiences bolstered her self-affirmation and empowered her to navigate unfamiliar settings in her distinctive way. The discussions within her counseling sessions became more frequent, and she progressively harnessed her inherent strength within her community and environment, eventually concluding her treatment without requiring constant therapist support (Table 1). The amount of medication used in the drug therapy was small and was only changed a few times. This is a common pattern in which medication is administered while conducting Morita therapy. The drug therapy was only provided as an adjunct to the Morita therapy [9]. Therefore, it was considered that this patient improved due to Morita therapy.

**Table 1:** Assessments, intervention, and therapist-patient relationship.

	First half	Second half
Assessments	- Operational diagnosis - Notification of a diagnosis - Co-occurring with dissociative hallucinations - Fragmented self-experience	- Unified self - A growth process that involves dealing with emotions and interacting with the environment
Intervention	- Isolation and rest from the environment - Adjustment and intervention in the environment - Promote a dedication to the environment through engagement in meaningful work	- Reflection on emotional experience - Psychotherapy using a diary (purification of emotional experience) - Exploring her own way of interacting with the environment
Therapist-patient relationship	- Maintaining a noninvasive and secure therapeutic relationship	- A genuine human relationship, incorporating humor - The therapeutic relationship extends directly to the patient’s world (nontransferable relationship)

Comprehending the psychopathological aspects of cases

**Figure 1:**
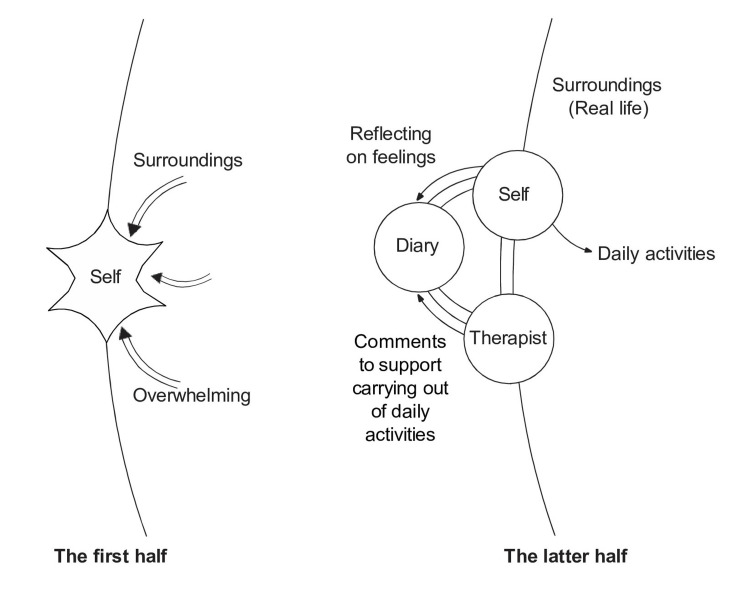
Structure of psychotherapy and relationship between environment and self. Image credit: Developed from a discussion between Dr. Kenji Kitanishi and Dr. Mitsuhiro Nakamura, November 15, 2023, at Morita Therapy Institute in Tokyo, Japan.

The implementation of Morita therapy becomes relevant as individuals acknowledge their struggle to accept themselves within their environments, a state Kora [6,7] refers to as *inadaptability anxiety*. Adolescents with ASD often perceive themselves as different from their peers, rendering day-to-day life challenging. Such circumstances are common in adolescents with ASD [10,11]. The core tenet of Morita therapy is for the therapist to bridge the gap between individuals who feel overwhelmed by their surroundings and the outside world, facilitating their reconnection with the broader world (Figure 1) [1,7,8].

At the outset of treatment, the patient grappled with impaired impulse control and emotional regulation, both of which could be attributed to her developmental characteristics and a history of childhood trauma [14,15]. Her parents, particularly her father, initially considered her as challenging to raise, finding her inscrutable due to her unique developmental traits. Regrettably, the parenting she received included instances of violence.

Furthermore, her traumatic experiences, including incidents of bullying during her elementary and junior high school years, later manifest in her adolescence as a fractured and bewildered sense of self, contributing to her emotional turmoil. Additionally, she endured auditory hallucinations that could be linked to her ongoing struggle to form a stable sense of self [13]. Despite her inability to distinctly articulate the reasons for her distress, she expressed a profound confusion and, at times, a desire to die. Such presentations are a commonly observed in adolescent wards dedicated to treating individuals with ASD. The self-experiences of young patients with severe ASD are often fragmented, lacking continuity in emotional experiences [10,11,13,14]. Grandin T., a prominent individual with PDD, likened his self-experience to a *library* containing many videotapes [16]. In Hirosawa’s [17] study, the self-images of adults with ASD were compared with those of neurotypical individuals, resulting in the characterization of what he termed the *touchscreen-like* self. Their emotional experience switch abruptly without continuity, resembling the way touching a touchscreen switches images. Although the origins of this experience are complex due to the physiological and neuroscientific background of ASD and attachment issues [14,15], it is noteworthy that fragmented selves and disjointed emotional experiences are common symptoms in many developmental disorders that emerge during adolescence. This observation is likely to resonate with clinicians working in the field.

In her diary entries, the patient eloquently described the experiences of her touchscreen self, perceiving certain interactions with others as *poisonous* and *deadly poisonous*. Thus, the intervention of Morita therapy has demonstrated its effectiveness in detoxifying and neutralizing such emotional toxins, equipping individuals with the capacity to detoxify themselves and neutralize threats.

Therapeutic intervention and the treatment process: the significance of diaries

In the initial stages of treatment of the patient, the focus was on maintaining an empathetic therapeutic relationship, including facilitating her admission to the adolescent ward as necessary to prevent crises. This involved actively engaging with her interests, laying the groundwork for implementing Morita therapy with the use of her diary [1,7,18]. Throughout our year-long therapeutic journey, she rarely verbalized her feelings in the examination room, often simply stating “I want to die.” Therefore, I suggested to her, “If you want to receive treatment properly, why not try using your diary?” - to which she agreed. Thus, diary therapy was introduced during her third hospitalization, marking the turning point in connecting with her fragmented self. Following the introduction of the diary, the therapist encouraged her to record her feelings of social anxiety and fear that arose during her hospital stay. She was also urged to participate in occupational therapy and adolescent group therapy, despite any resulting anxiety. There was a pivotal moment when her adolescent group accepted her self-affirmation regarding her uncertainty about her sexual orientation.

Upon her discharge from the hospital, she was encouraged to establish social connections in areas where she excelled. Through introspection to discern her healthy sensibilities and unfiltered desires, she gradually became more engaged in real-life interactions within her realms of expertise, ultimately finding her place in the world.

In cases of adolescent ASD, despite the intermittent and sometimes perplexing nature of the themes and the patient’s unconventional actions and choices, a bond of trust gradually develops between the doctor and the adolescent. Over time, the focus of the treatment journey shifts from addressing the symptoms to exploring ways of living authentically, encompassing the acceptance of one’s gender and unique characteristics. This transformative process culminates in a completed treatment.

This evolution necessitates the establishment of a consistent and empathetic therapeutic relationship, which may involve the judicious use of hospitalization when necessary to avert crises, thoughtful assessment of the patient’s capacities and limitations in different life situations, and the acquisition of the skills needed to navigate life in the presence of anxiety. This holistic approach enables the effective treatment of adolescent patients with ASD while fostering their personal growth simultaneously.

Notably, a remarkable contrast is evident between the initial entry in Diary 1, at the start of her hospitalization, where she uniquely expressed her social anxiety, and Diary 3, toward the conclusion of her treatment. In the latter entry, she demonstrates her strengths and embraces her individuality.

Contemporary clinical practices for ASD, which emphasize *environmental adjustment*, often do not directly facilitate the development of adolescent patients with ASD as described here [8]. While numerous psychotherapeutic approaches have been explored and documented for ASD, the form of psychotherapy that profoundly influences the growth journey of individuals with developmental disabilities, as advocated by Morita, extends beyond the confines of the therapy room and into the individual’s everyday living environment [1].

Instead of regarding ASD as a *hindrance* to social communication, it is crucial to foster the capacity to empathize with and navigate occasionally distressing interactions with others, experiences that can be described as *poisonous* or *deadly poisonous*. This approach also entails addressing positive emotions and emotional sensitivity.

When implemented with adolescents, Morita therapy can serve as a growth model and can contribute not only to the development of individuals with typical development but also to those with ASD and other developmental disorders [9,18].

Aspects of the therapist-patient relationship: mutual exchange, shared self-disclosure, and a collaborative model for mutual growth

The patient’s doctor, initially on the outskirts of the realm of the *deadly poisonous* and *poisonous* relationships with others, diligently worked to maintain a nonintrusive and empathetic therapeutic relationship with the patient. Despite initial challenges with introducing diary therapy, progress was made, and before the patient’s third hospitalization, she and her doctor successfully integrated diary therapy into her treatment, building upon the foundation of the therapeutic rapport that had been established. Together, they embarked on a journey of self-expression through the patient’s unique style of diary writing.

Throughout this process, the doctor aimed to establish a therapeutic alliance centered on finding a *detoxification method*, which proved pivotal in the patient’s treatment. The doctor was genuinely impressed by the vibrant and distinctive sensibility that the patient conveyed in her diary entries, providing candid feedback during their examination sessions as well as through comments on her diary writings.

Furthermore, the doctor observed that the patient found enjoyment in activities such as cosplay and dancing. Self-psychology, a Western academic and clinical framework centered on concepts of *empathy* and the *self*, was founded by Kohut as a departure from classical psychoanalysis, where clinicians assumed a blank screen role [13,19,20]. As self-psychology evolved, empathy came to be recognized as a bidirectional process [13,20].

Along the therapeutic journey, my exploration of my own adolescent psyche, at times involving self-disclosure, proved instrumental in practicing Morita therapy. Notably, by the end of the treatment, the therapeutic relationship transcended the bounds of transference and seamlessly merged with daily life, signifying the successful completion of the treatment [18]. Reflecting on this historical context, in 1919, when Shoma Morita initiated inpatient treatment for a case involving blushing, he refined his sensibilities and evolved as a therapist [18,20]. Similarly, during the five years of the unfolding of this case, Morita therapy not only facilitated a remarkable recovery from severe ASD but also nurtured the clinician’s Morita therapeutic skills and clinical acumen related to adolescent ASD.

## Conclusions

For adolescents, Morita therapy presents a growth-promoting model adaptable to ASD. In this case, based on Morita therapy, we were able to understand from the anxiety and fear the raw desire that was driving them and treat them all with the use of a diary, contributing to the growth of the patient’s characteristics and the disappearance of the symptoms of dissociation, self-injury, and auditory hallucinations. In treating adolescents who have both severe ASD and other psychiatric symptoms including self-destructive behaviors and psychotic symptoms, it is important for the therapist to fully empathize with the fears and anxieties that the patient has while encouraging the discovery of the patient’s healthy abilities and their expression.

This treatment course requires an extended period of psychotherapy in two phases: a preparatory phase and an interactive, psychotherapeutic phase. If the patient has some linguistic ability, a therapeutic dialogue in the form of a diary can be very useful. Here, according to the principles of Morita therapy’s use of a diary, the diary effectively encouraged the expression and acceptance of emotional experiences and enabled the patient to comment on her health in light of her daily activities. As a result of this approach, psychotic symptoms such as auditory hallucinations and impulsivity subsided. Even if Morita therapy is not used, it is necessary to treat ASD by not only adjusting the environment but also by addressing the patient’s anxieties and fears. Moreover, it is important to take a long-term view over a period of several years, using hospitalization and other forms of rest from the environment, as appropriate, to prevent crisis.
